# A Scoping Review of Person-Centered Mental Health Care for Migrants and Refugees in Humanitarian Settings

**DOI:** 10.1177/00207640261473294

**Published:** 2026-08-03

**Authors:** Desalegn Melika Geleta, Myung-Bae Park, Eunwoo Nam, Nancy Clark

**Affiliations:** 1 Yonsei Healthy City Research Center, Wonju, Gangwon-do, South Korea; 2 Yonsei University Mirae Campus, Wonju, Gangwon-do, South Korea; 3 University of Victoria, BC, Canada

**Keywords:** migrants, refugees, person-centered care, mental health, scoping review, humanitarian settings

## Abstract

Refugees and migrants experience disproportionately high rates of depression, anxiety, and post-traumatic stress disorder due to pre-migration trauma and post-resettlement challenges. Although the World Health Organization (WHO) QualityRights framework emphasizes person-centered and rights-based mental health care, its practical application in migrant and refugee mental health settings remains underexplored. This scoping review maps existing evidence on person-centered mental health care (PCC-MH) for migrants and refugees, identifies key barriers and facilitators, and describes existing interventions and care models through the lens of the WHO QualityRights framework. Following PRISMA-ScR guidelines, six academic databases and relevant gray literature sources were searched for studies published between 2004 and 2025. Included studies addressed PCC domains such as dignity, autonomy, communication, privacy, stigma reduction, and cultural sensitivity. Data were synthesized and interpreted using the WHO QualityRights framework. Forty-four studies met the inclusion criteria. The evidence consistently showed high mental health needs among migrants and refugees alongside low service utilization. Key barriers included language difficulties, stigma, restrictive policies, and limited cultural competence within health systems. Facilitators comprised culturally adapted interventions, interpreter services, peer- and trauma-informed care, and rights-based approaches, though implementation was often fragmented and inconsistent. Achieving equitable and sustainable mental health care for migrants in humanitarian settings requires systematic integration of PCC principles within national mental health policies and primary-care systems. Core PCC principles must include privileging the rights, lived experience, and voice of migrant groups to effectively promote the mental health and well-being of diverse migrant groups in humanitarian settings. This can include co-designing mental health services and supporting migrants.

## Background

Migration and displacement are among the defining global challenges of the 21st century. In 2023, the United Nations High Commissioner for Refugees (UNHCR) reported that over 110 million people worldwide were forcibly displaced due to conflict, persecution, environmental crises, and socioeconomic hardships. These numbers are steadily rising, with approximately 123.2 million people needing international protection due to war, human rights violations, climate emergencies, and political persecution (UNHCR, 2025).

Refugees, asylum seekers, and other displaced populations collectively referred to as migrants (International Organization for Migration [IOM], 2019) experience disproportionately high levels of psychological distress, depression, anxiety, and post-traumatic stress disorder (PTSD) stemming from pre-migration trauma, perilous journeys, and post-migration stressors such as acculturative difficulties, unemployment, discrimination, and insecure legal status (Fazel et al., 2005). A recent systematic review suggests that PTSD and depression for refugees and asylum seekers are greater than in the general population, at approximately 31% and 31.5%, respectively (Blackmore et al., 2020). In humanitarian settings such as Sheder Refugee Camp in southeastern Ethiopia, high prevalence rates of common mental health problems such as depression (41.1%), generalized anxiety disorder (55.0%), substance use disorder (49.3%), and mood disorders (41.4%) were observed among adult camp residents, with nearly all (98%) reporting traumatic experiences, highlighting the fact that migrants both pre- and post-migration are at elevated risk for mental health problems and underscoring the urgent need for culturally responsive mental health services (Desalegn, 2025). Despite growing awareness of the mental health burden among migrants, the quality and PCC they receive remain insufficiently explored.

Person-centered care (PCC) has emerged as a global standard for improving health system responsiveness and equity. However, the concept has not been widely applied in post-migration contexts such as humanitarian settings.Humanitarian settings are created in response to conflict and natural disasters and often raise human rights concerns (UNHCR, 2025). These settings thus align with the need for what the World Health Organization defines as PCC, as care that is respectful of and responsive to individual patient preferences, needs, and values, ensuring that these guide all clinical decisions (WHO, 2018). Responsive mental health is reinforced through PCC frameworks such as the WHO QualityRights initiative, which calls for services that uphold dignity, autonomy, informed decision-making, participation, and freedom from coercion (Citaristi, 2022).

Forcibly displaced migrants residing in humanitarian settings face several barriers to person-centered mental health care (PCC-MH). Language differences hinder effective communication and can lead to misdiagnoses and poor adherence to treatment (Brandenberger et al., 2019). Cultural variations in the conceptualization of mental illness can conflict with biomedical models, undermining trust in services (Kirmayer & Ryder, 2016). Structural barriers including costs, restrictive policies, insecure residency status, and discrimination, further limit access to dignified care (Silove et al., 2017). Stigma, both within migrant communities and host societies, remains a pervasive deterrent to seeking help (Satinsky et al., 2019).

Community-based and culturally adapted interventions such as peer-navigator programs, trauma-informed counseling, and culturally tailored cognitive behavioral therapies have improved mental health outcomes and patient experiences among displaced populations, particularly in higher-income contexts (Hinton & Jalal, 2014). Integrating interpreters and cultural mediators into mental health services can enhance communication, foster trust, and mitigate stigma for migrants (Bischoff & Hudelson, 2010). Most systematic reviews of migrant mental health have focused on prevalence, service utilization, and barriers to access in resource-rich countries (Priebe et al., 2016; Satinsky et al., 2019). Furthermore, most research focuses on deficits of persons living with a mental illness rather than strengths-based perspectives. Consequently, there is limited understanding of how care is delivered and whether it adheres to person-centered principles such as dignity, autonomy, privacy, and cultural sensitivity in humanitarian settings.

Given the extent of global displacement and the central role of mental health in integration, resilience, and overall well-being, understanding PCC approaches in migrant mental health is both urgent and timely. Mapping how PCC has been conceptualized, implemented, and evaluated across migrant mental health settings can generate critical insights for clinicians, policymakers, and humanitarian actors seeking to uphold human rights, dignity, and strengths-based approaches to care.

PCC explicitly interrogates not only whether care is accessible, but how it is delivered and experienced, particularly among marginalized populations. For migrants and refugees whose interactions with health systems are often shaped by legal precarity, discrimination, and cumulative trauma, PCC provides an essential lens for assessing the acceptability, legitimacy, and ethical grounding of mental health services. Ultimately, engagement with QualityRights-informed PCC approaches may support the development of more culturally responsive models of care and policies that uphold dignity and human rights, irrespective of legal status or country of origin. This review, therefore, aimed to inform future PCC-oriented mental health policies and culturally sensitive care models for forcibly displaced populations worldwide.

## Methods and Materials

This study utilized the Preferred Reporting Items for Systematic Reviews and Meta-Analyses Extension for Scoping Reviews (PRISMA-ScR) to guide reporting of findings. The scoping review followed the standard steps of familiarization with existing literature, systematic searching, screening, data extraction, and data analysis, as outlined by Arksey and O’Malley (2005) and elaborated by Levac, Colquhoun, and O’Brien (Powell et al., 2020). The World Health Organization (WHO) QualityRights framework was used as the primary analytical lens for data synthesis, guiding the identification, categorization, and interpretation of PCC-MH domains across the included studies (WHO, 2019–2023, 2023b, 2024).

### Step 1: Identifying the Research Question

This scoping review searched the literature on PCC-MH in programs and services targeting migrants, refugees, asylum seekers, and other displaced populations in humanitarian and host-country settings. The review aimed to map existing evidence, study types, and research gaps, while providing a broad overview of the role of PCC in migrant mental health. We synthesized the literature to describe PCC-MH, critically analyze the delivery of mental health services, and conceptualize person-centered approaches in both humanitarian and host country contexts. The study addressed the following research questions:

1. What evidence exists regarding PCC-MH for migrants and refugees in humanitarian and host country settings?
2. What are the barriers and facilitators to implementing PCC-MH in these populations?
3. What interventions or models of care have been implemented to enhance person-centered mental health services for migrants and refugees?

### Step 2: Identifying Relevant Studies

The search strategy was developed iteratively and applied across PubMed, PsycINFO, MEDLINE, EMBASE, Scopus, and Web of Science using a combination of controlled vocabulary and free-text terms related to migration, mental health, and person-centered care. The review covered studies published between 2004 and 2025, reflecting the emergence of person-centered care as a recognized framework in global health and mental health policy in the early 2000s, alongside the growing emphasis on rights-based approaches to care. The final search strategy was adapted for each database to account for differences in indexing and syntax.

All retrieved records were imported into EndNote 25, and duplicates were identified and removed before screening. After duplicates were removed, title and abstract screening were conducted independently by two reviewers, followed by a full-text review to confirm eligibility. A standardized data extraction form was used to capture the study characteristics (author, year, country, design, and population), PCC-MH domains addressed, reported outcomes (experience, satisfaction, and barriers/facilitators), and any interventions or models of care. Two reviewers independently conducted title/abstract and full-text screening, resolving disagreements through discussion and consensus. If they couldn’t agree, a third reviewer was consulted.

### Step 3: Selecting Studies to be Included in the Review

We applied a Population, Concept, Context, Outcome (PCCO/PICO-like) framework to guide the study selection.

1. Population: Migrants, refugees, asylum seekers, displaced people, and relevant mental health providers.
2. Concept: PCC-MH, including domains such as dignity, autonomy, privacy, communication, confidentiality, cultural sensitivity, stigma reduction, and supportive care.
3. Context: Humanitarian settings (e.g., refugee camps, post-conflict zones) and host-country mental health services.
4. Outcome: Evidence related to experiences, perceptions, satisfaction, barriers and facilitators, implementation strategies, and interventions for PCC-MH.

Evidence published from 2004 onward was included, reflecting the growing global focus on PCC in healthcare. The final search was completed in 2025. Only studies written in English were included, although relevant gray literature from international organizations was considered. The scoping review methodology permits the inclusion of all study designs, including quantitative, qualitative, and mixed-methods studies, as well as program reports and evaluations. Brief reports, conference abstracts, preprints, or opinion pieces were excluded.

### Step 4: Charting Data

The eligibility of the studies was independently screened by all researchers conducting the scoping review. Overall, the included studies demonstrated moderate to high methodological quality.

Data from the final selected articles were extracted. Extracted information included:

1. Basic study characteristics: author, publication year, country, study design
2. Population characteristics: migrants, refugees, asylum seekers, mental health providers
3. Type of mental health program or service, aim, and evaluation design/methods
4. Key findings relevant to PCC-MH domains (dignity, autonomy, communication, privacy, confidentiality, stigma reduction, supportive care, and cultural sensitivity)
5. Contextual factors influencing PCC-MH implementation
6. Major conclusions and implications for practice.

### Step 5: Collating, Summarizing, and Reporting the Results

Data were synthesized narratively, with findings mapped onto the PCC-MH framework and WHO quality of care domains. The findings were presented based on the PCC-MH domains of dignity, communication, autonomy, privacy, confidentiality, supportive care, and cultural sensitivity, aligned with the WHO QualityRights framework. Best practices and innovative interventions promoting PCC-MH were also highlighted. Tables and figures summarize the study characteristics, geographic distribution, PCC-MH domains, and key barriers and facilitators. Table 1 details the inclusion and exclusion criteria for the studies included.

**Table 1.** Inclusion and Exclusion Criteria to Assess PCC-MH for Migrants and Refugees.

PICO	Inclusion criteria	Exclusion criteria
Population	Migrants, refugees, asylum seekers, displaced people, and mental health providers of any age	Populations from non-humanitarian settings, the general population without a migration context
Intervention / Concept	Approaches, programs, or strategies targeting PCC-MH, including trauma-informed care, counseling, psychotherapy, community-based interventions, peer support, and cultural adaptation. Person-centered mental health care (PCC-MH), operationalized using WHO QualityRights domains (dignity, autonomy, participation, communication, privacy, cultural sensitivity)	Interventions not related to mental health or PCC
Comparison	Studies exploring patient or clinician perspectives on PCC-MH, experiences with services, or evaluations of interventions designed to enhance PCC-MH	Studies without a focus on PCC-MH principles or experiences
Outcome	Awareness, experience, perception, satisfaction with care, dignity, autonomy, privacy, communication, stigma reduction, cultural sensitivity, supportive care, and engagement in care	Outcomes unrelated to PCC-MH
Publication Year	Articles published from 2004 onward	Articles published before 2004
Language	English (working language of the review team)	Other languages
Setting	Humanitarian and host-country settings, including refugee camps, health facilities, and community-based services	Non-humanitarian, general population, or private clinic settings
Type of Study	Quantitative, qualitative, or mixed-method studies describing PCC-MH dimensions or implementation	Brief reports, brief communications, pre-prints, conference abstracts, presentations

### Patient and Public Involvement

No patients or members of the public were involved in this study.

## Results

### Study Selection

A total of 65 records were identified through database searching, and 15 additional records were identified through citation tracking and manual searching. After removing duplicates, 50 articles underwent title and abstract screening. Following full-text review, 44 studies met the inclusion criteria. A PRISMA-ScR flow diagram illustrating the selection process is shown in Figure 1.

**Figure 1.**
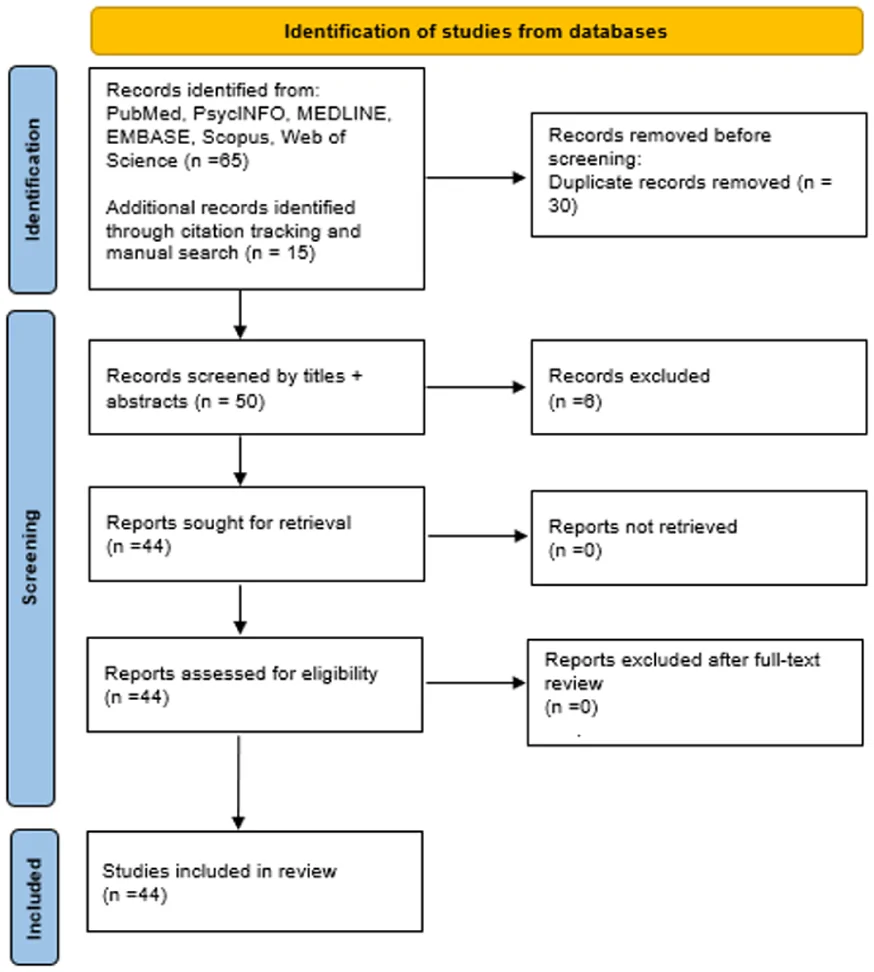
PRISMA-ScR flow diagram of studies included in the review.

### Characteristics of Included Studies

The included studies (*n* = 44) published from 2004 to 2025. A summary of the included studies is shown in Table 2.

**Table 2.** Characteristics of the Included Studies in the Review.

No.	Author(s)	Year	Country/region	Study design	Population/setting	Key PCC-MH relevance
1	Abayneh et al.	2018	Global	Realist systematic review protocol	Service users, caregivers, mental health systems	Focus on integrating user and caregiver involvement for system strengthening directly relevant to person-centered approaches.
2	Altahsh et al.	2025	Canada	Qualitative; Nominal Group Technique	Refugee Patient Advisory Committee, Calgary	Empowers refugee voices to set priorities illustrates participatory PCC methods.
3	Betancourt et al.	2013	Multi-region; war-affected	Ecological review on interventions	Children affected by war	Emphasizes ecological, system-level PCC for child mental health in conflict settings.
4	Bischoff and Hudelson	2010	Switzerland	Qualitative / communication study	Migrant patients and clinicians	Highlights interpreter access and provider training critical for culturally safe PCC.
5	Blackmore et al.	2020	Global	Systematic review and meta-analysis	Refugees and asylum seekers	Quantifies high prevalence of mental illness; underscores urgent need for PCC-oriented services.
6	Brandenberger et al.	2019	High-income countries	Systematic literature review	Migrants and refugees in HIC health systems	Introduces 3C model; identifies barriers to effective PCC delivery.
7	Canda	2024	Global / conceptual	Transcultural perspective; conceptual chapter	Social work with cultural and spiritual diversity	Stresses cultural and spiritual dimensions key to person-centered, culturally responsive care.
8	Citaristi	2022	Global directory context	Organizational description	UNHCR and related international organizations	Provides context on global structures; useful background for PCC policy framing.
9	Desalegn	2025	Ethiopia	Cross-sectional assessment	Refugee camp residents	Empirical data on common mental health problems; supports local relevance of PCC.
10	Fazel et al.	2005	UK, USA, Australia	Systematic review	Resettled refugees	Early evidence of serious mental disorder prevalence; foundational for PCC advocacy.
11	Henley et al.	2011	Humanitarian contexts	Case study evaluation	Humanitarian relief settings	Demonstrates integration challenges continuity of PCC in emergencies.
12	Hinton et al.	2014	Global	Implementation guidelines	Refugee therapy settings	Offers culturally sensitive CBT guidance practical PCC adaptation.
13	Hynie et al.	2023	Canada and global evidence	Scoping review	Refugee and immigrant groups	Access to virtual mental healthcare; informs PCC in digital/remote settings.
14	Im et al.	2023	United States	Implementation evaluation	Community-based mental health and psychosocial support for refugee newcomers	Shows real-world rollout issues and PCC implications for community programs.
15	IOM (Ed.)	2019	Global	Reference glossary	Migration field	Defines key terms; background resource for PCC discussion in migration context.
16	Jarvis et al.	2020	Global	Update on Cultural Formulation Interview	Clinical practitioners	Enhances structured cultural assessment tool for PCC practice.
17	Kirmayer and Jarvis	2019	Global	Conceptual / commentary	Health services	Argues culturally responsive services for equity aligns with PCC goals.
18	Kirmayer and Ryder	2016	Global	Narrative review	Cross-cultural populations	Links culture and psychopathology; supports culturally anchored PCC models.
19	Kronick et al.	2021	Global	Conceptual chapter	Refugee mental health and human rights	Frames PCC within human rights obligations.
20	Lau and Rodgers	2021	Global	Scoping review	Refugee service settings	Synthesizes cultural competence evidence—an enabler of PCC.
21	Lustig et al.	2004	USA and broader	Review	Child and adolescent refugees	Calls for family/system approaches child-centered PCC emphasis.
22	Walpola et al.	2022	Global	Systematic review	Refugees and asylum seekers in primary care	Examines interventional approaches to improve PCC quality.
23	Meherali et al.	2024	South Asian diaspora contexts	Qualitative / narrative	South Asian immigrant youth	Identifies challenges in transnational contexts; informs PCC adaptation for youth.
24	Mezzina	2025	Global	Conceptual / commentary	Mental health services	Discusses innovations in service delivery context for PCC evolution.
25	Mezzina et al.	2018	Global	Conceptual synthesis	Human rights and global mental health	Positions PCC as practice of freedom; rights-based framework.
26	Miller et al.	2006	Afghanistan conflict zone	Tool development	Conflict-affected adults	Afghan Symptom Checklist; culturally grounded assessment tool for PCC.
27	Negash et al.	2025	Humanitarian and fragile settings	Scoping review	Maternity care services	Person-centered care lessons transferable to mental health settings.
28	Olcoń et al.	2023	Australia	Implementation gaps study	Refugee and migrant maternal health	Highlights gaps in culturally responsive care relevant for PCC systems.
29	Orang et al.	2023	Europe or global	Randomized controlled trial	Migrants and refugees	Tests Value Based Counseling; evaluates culturally sensitive PCC intervention.
30	P. Iqbal et al.	2022	Global	Systematic review	Refugees and asylum seekers in primary health care	Similar to entry 23; reinforces evidence on intervention effectiveness for PCC.
31	Patel et al.	2018	Global	Commission report	Global mental health systems	Calls for PCC within wider sustainable development agenda.
32	Poudyal and Adhikari	2022	Low- and middle-income countries	Review or synthesis	Mental health in LMICs	Identifies needs, gaps, and recommendations underpins PCC policy relevance in LMICs.
33	Powell et al.	2020	Global or USA	Scoping review	Family caregivers of women/girls with intellectual disability	While outside refugee focus, highlights caregiver role insight for PCC caregiving models.
34	Priebe et al.	2016	WHO European region	Policy and evidence review	Refugees, asylum seekers, irregular migrants	Recommends rights-based, person-centered care; directly informs PCC guidance.
35	Satinsky et al.	2019	Europe	Systematic review	Refugees and asylum seekers	Shows low utilization of care; advocates improving PCC to address access barriers.
36	Silove et al.	2017	Global	Overview review	Refugees and asylum seekers	Contextualizes trauma and stressors; supports need for PCC frameworks.
37	Tol et al.	2015	Humanitarian settings	Public mental health perspective review	Populations in crises	Connects public health and PCC principles in humanitarian MHPSS.
38	UNHCR	2025	Global	Data report	Refugees, migrants	Global Trends report; situational context for PCC policy planning.
39	Weissbecker et al.	2018	Humanitarian crisis settings	Program review or synthesis	Refugee populations	Integrative MHPSS interventions align with PCC ideals.
40	WHO	2018	Global	Implementation framework	Older populations	ICOPE framework; general guidance relevant for PCC across populations.
41	WHO	2019–2023	Global	Action plan	Refugee and migrant health	Global action plan; policy context supporting PCC approaches.
42	WHO	2023a	Global	Program budget report	Health programs	Operational context; resource prioritization for PCC-related initiatives.
43	WHO	2023b	Global	Technical or thematic report	Refugees and migrants	Risk/protective factors and access; directly relevant for PCC planning.
44	WHO	2024	Global	Report or narrative	Population health	Exposure of achievements; reinforces PCC in service access stories.

### Burden of Mental Disorders

Point prevalence varies by disorder, study population, and setting. Post-traumatic stress and depressive disorders remain the most reported conditions. Studies conducted in humanitarian camps and LMIC settings have also reported particularly high levels of depression and anxiety, with findings linked to ongoing material deprivation, insecurity, and protracted displacement (Tol et al., 2015). Importantly, the burden of mental risks and conditions is compounded even after displaced migrants have been granted resettlement outside of conflict zones. Despite such a high prevalence, service utilization is strikingly low in both humanitarian and resettlement settings. Migrant groups, refugees, and asylum seekers frequently face barriers to accessing mental health care, including legal and structural restrictions, language and cultural barriers, stigma, and a lack of culturally competent providers (Abayneh et al., 2018; Altahsh et al., 2025; Im et al., 2023; Olcoń et al., 2023). Reviews from Europe and high-income settings show a consistently low uptake of formal mental health services despite mental health promotion efforts signaling person-centered care approaches (Bischoff & Hudelson, 2010; Brandenberger et al., 2019; Satinsky et al., 2019).

### Barriers to and Facilitators of Accessing Mental Health Care in Humanitarian Settings

Comparatively, structural barriers (restrictions linked to asylum status, limited entitlement to public services, and high out-of-pocket costs in both high-income host countries and humanitarian settings) were most consistently reported (Priebe et al., 2016; Satinsky et al., 2019). Health systems in humanitarian settings often face underfunding and overload, lacking sufficient trained staff, medications, and psychosocial support infrastructure (Brandenberger et al., 2019; Silove et al., 2017).

Moreover, language and communication barriers were pervasive across LMICs and HICs. Studies have shown that even when professional interpreters are available, communication quality is often compromised, undermining trust and limiting the accuracy of assessments (Bischoff & Hudelson, 2010). The absence of culturally adapted services and reliance on Western diagnostic categories in HIC Western contexts can further alienate patients, particularly when expressions of distress do not fit biomedical models (Kirmayer & Ryder, 2016). Thus, it is not surprising that similar barriers were reported in humanitarian contexts where resources are constrained and where migrant groups may experience multiple forms of discrimination.

Mental health stigma, within migrant communities and non-migrant communities in host societies, represents another powerful barrier. Mental illness is frequently associated with shame, fear of discrimination, or concerns about jeopardizing asylum claims, which discourages help-seeking (Fazel et al., 2005; Hinton & Jalal, 2014). For migrant, racialized women, stigma is often compounded by gender-based violence, restrictive gender norms, and limited autonomy in healthcare decision-making (Negash et al., 2025).

Facilitators for promoting PCC included community-based and culturally adapted interventions for example, adapted cognitive behavioral therapy (CBT) that bridge cultural divides and reduce stigma (Hinton & Jalal, 2014). World Health Organization Global Action Plan “Promoting the health of refugees and migrants” (2019–2023) and the WHO “World Mental Health Report: Transforming health for all,” person-centered care have highlighted as favoring the voices and lived experiences of those affected by social determinants of mental health, that is, war and displacement (WHO, 2018, 2019–2023).

These policy and practice interventions promote continuity of care, trust with providers, and can destigmatize the construction of mental health. Increasing treatment retention and adherence also when PCC is promoted through services that help mitigate barriers to migrant mental health care (Brandenberger et al., 2019; Negash et al., 2025).

### Cultural and Linguistic Sensitivity

Cultural and linguistic sensitivity has emerged as a critical aspect of PCC-MH in both humanitarian and host country settings. Across the included studies, culturally and linguistically adapted approaches were associated with improved engagement, adherence, and mental health outcomes among refugees and migrants (Fazel et al., 2005; Hinton & Jalal, 2014; Priebe et al., 2016). However, implementation is often challenged by language barriers, limited provider training, and structural constraints within healthcare systems (Bischoff & Hudelson, 2010; Brandenberger et al., 2019).

Numerous studies highlight the need for culturally grounded assessment tools and interventions. For instance, the Afghan Symptom Checklist provides a culturally specific measure of psychological distress for conflict-affected populations, demonstrating that standard instruments may fail to capture culturally meaningful expressions of mental health problems (Miller et al., 2006). Guidelines for culturally sensitive CBT further highlight the adaptation of therapeutic content, metaphors, and delivery methods to align with client cultural frameworks (Hinton & Jalal, 2014). Culturally sensitive interventions, including value-based counseling, enhance engagement and reduce dropout rates while respecting clients’ cultural identity and autonomy (Meherali et al., 2024). Child and adolescent populations particularly benefit from interventions that integrate family systems, local explanatory models, and cultural norms (Betancourt et al., 2013; Lustig et al., 2004). Besides psychologically tailored interventions, relational adaptations and behavioral change of providers to practice cultural safety have also shown to mitigate stigma, discrimination, and barriers to mental health services and supports (Lau & Rodgers, 2021).

Culturally informed frameworks such as cultural safety and the cultural formulation tool can help distinguish between normative cultural expressions of distress and clinical psychopathology, thereby reducing misdiagnosis and facilitating person-centered care (Kirmayer & Jarvis, 2019; Kronick et al., 2021). Ecological models of refugee distress further highlight the influence of community and sociocultural factors on mental health outcomes. Less is known about how these approaches are integrated within humanitarian settings, where migrants experience higher levels of psychological distress (Jarvis et al., 2020).

Language barriers significantly hinder effective mental healthcare. Access to professional interpreters improves diagnostic accuracy, therapeutic alliances, and patient satisfaction (Bischoff & Hudelson, 2010; Satinsky et al., 2019). However, interpreter access alone is insufficient; clinicians require training to recognize subtle linguistic nuances and culturally embedded expressions of distress (Brandenberger et al., 2019; Hinton & Jalal, 2014). Digital interventions, such as telepsychiatry and mobile applications, show promise for addressing linguistic and access barriers, particularly for populations in humanitarian settings. Nevertheless, their effectiveness depends on culturally and linguistically adapted content and interfaces (Satinsky et al., 2019).

Despite its recognized importance, implementing culturally and linguistically sensitive care remains challenging. Common barriers include the limited availability of trained personnel, inadequate institutional policies, and inconsistent integration of cultural competence into standard protocols (Brandenberger et al., 2019; Patel et al., 2018). Refugees and migrants often report experiences of miscommunication, mistrust, and perceived discrimination, which can discourage their engagement in mental health services (Satinsky et al., 2019).

Facilitators of culturally responsive care include adherence to the WHO’s person-centered and rights-based guidance (WHO, 2023a, 2023b). Emphasizing community engagement and co-designed interventions with refugee populations as necessary to uphold strengths-based approaches and cultural safety (Negash et al., 2025). Overall, education and Training programs can improve providers’ cultural competence, knowledge, and sensitivity so that they may advocate for integration of professional interpreter services; these have been shown to strengthen therapeutic relationships, build trust, and improve mental health outcomes for migrant groups (Bischoff & Hudelson, 2010; P. Iqbal et al., 2022).

Table 3 summarizes the key intervention categories identified across the included studies that aim to enhance PCC-MH for migrants and refugees. These interventions span provider-level, community-based, and system-level strategies, reflecting efforts to address interpersonal care practices alongside structural and policy determinants influencing the delivery of PCC-MH in humanitarian and host-country settings.

**Table 3.** Summary of Person-Centered Mental Health Care (PCC-MH) Interventions for Migrants and Refugees, Mapped to WHO QualityRights Domains.

Category	Interventions
Provider training and support	Training programs on cultural competence and trauma-informed care; integration of the WHO QualityRights framework into provider practice, supervision, and ongoing capacity building (Brandenberger et al., 2019; Kirmayer & Jarvis, 2019; Lau & Rodgers, 2021; Mezzina et al., 2018; Patel et al., 2018; WHO, 2024).
Language support	Professional interpreters, use of cultural mediators, development of linguistically adapted tools (Bischoff & Hudelson, 2010; Hynie et al., 2023; Miller et al., 2006; Satinsky et al., 2019; Weissbecker et al., 2018).
Cultural and social support	Culturally adapted CBT, value-based counseling, family- and community-based interventions for children and adolescents, peer navigator/peer support programs (Betancourt et al., 2013; Hinton & Jalal, 2014; Lustig et al., 2004; Meherali et al., 2024; Orang et al., 2023).
Integration and financial coverage	Integration of mental health into primary health care, rights-based approaches to ensure equitable access irrespective of legal status (P. Iqbal et al., 2022; Patel et al., 2018; Poudyal & Adhikari, 2022; Satinsky et al., 2019; Tol et al., 2015).
Community engagement	Co-design of services with refugees; community-based psychosocial interventions; involvement of local leaders, mediators, and peer-support workers (Abayneh et al., 2018; Altahsh et al., 2025; Hynie et al., 2023; Negash et al., 2025; WHO, 2023a).
Policy and system-level actions	Embedding PCC into policies and guidelines, digital/telehealth interventions with cultural adaptation, inter-sectoral collaboration (government–NGO–UN; Henley et al., 2011; Hynie et al., 2023; Mezzina, 2025; Priebe et al., 2016; WHO, 2018, 2019–2023, 2024).
Human-rights- and dignity-based frameworks	Rights-based interventions protecting autonomy, privacy, dignity, and freedom from coercion; participatory approaches promoting empowerment and agency (Kronick et al., 2021; Mezzina et al., 2018; Orang et al., 2023; Patel et al., 2018; WHO, 2023a, 2024).

### Human Rights and PCC Frameworks

Human rights principles underpin the delivery of PCC-MH for migrants and refugees. Across the studies included, integrating rights-based frameworks was associated with greater dignity, autonomy, and equitable access to mental health services. Several international guidelines emphasize that mental healthcare should not only address clinical needs but also respect legal, social, and cultural rights, particularly for forcibly displaced populations (Mezzina et al., 2018; WHO, 2023a).

A human rights approach emphasizes the protection of autonomy, dignity, privacy, and freedom from coercion in mental health care. Interventions that actively incorporate these principles, such as the practice of the freedom model, promote empowerment and agency among refugees and foster trust and engagement with services (Mezzina, 2025; Mezzina et al., 2018). Similarly, WHO (2023a) guidance on community mental health services advocates care that is rights-based, non-discriminatory, and responsive to individual needs, emphasizing participation and inclusion (WHO, 2023a).

PCC frameworks closely align with human rights principles because they focus on respecting individuals’ values, preferences, and expressed needs and voices. Effective PCC can involve co-designing services for migrants, incorporating culturally and contextually relevant interventions that privilege migrant knowledge about their lived experiences (Negash et al., 2025; Patel et al., 2018). PCC frameworks have the potential to improve therapeutic relationships, patient satisfaction, and positive mental health outcomes. Importantly, centering PCC within a human rights approach can promote bottom-up advocacy and address structural inequities and social determinants of health (Meherali et al., 2024).

Several models illustrate the internationalization of rights-based PCC in humanitarian and host-country settings. For example, value-based counseling integrates cultural sensitivity, human rights principles, and participatory approaches to care, leading to better engagement and perceived dignity among service users (Orang et al., 2023). Similarly, the WHO’s person-centered mental health implementation guide provides practical steps to embed rights-based principles into service delivery, emphasizing assessment, planning, and monitoring in collaboration with communities (WHO, 2024).

Despite international guidance, implementing rights-based PCC remains challenging. Structural constraints such as under-resourced health systems, a shortage of trained personnel, and inconsistent compliance with human rights frameworks limit the realization of PCC principles (Brandenberger et al., 2019; Patel et al., 2018). Additionally, systemic discrimination, legal restrictions, and a lack of political will can prevent equitable access to mental health services for migrants and refugees (P. Iqbal et al., 2022; Patel et al., 2018)

Facilitators of rights-based PCC include adherence to international guidelines, integration of legal and ethical standards into mental health protocols, training in human rights for providers, and participatory service designs for affected populations (Negash et al., 2025; WHO, 2023a). Owing to the impacts of mental health stigma across other social dimension categories such as migrant, gender, and ethnicity, ensuring privacy, confidentiality, and informed consent were repeatedly identified as critical elements of effective rights-based PCC (WHO, 2023a).

### Policy/System-Level Interventions, Guided by WHO Frameworks (2018) and PCC Implementation Models

Policy- and system-level interventions are critical for implementing PCC-MH for refugees and migrants. The WHO frameworks, including the Integrated Care for Older People (ICOPE) framework and PCC implementation guides, provide structured approaches to integrate rights-based, culturally sensitive, and sustainable mental health services across humanitarian and host country settings (WHO, 2018, 2019–2023).

System-level strategies include embedding PCC principles in organizational policies, clinical protocols, service delivery models, training personnel, and monitoring service quality. Integrating mental health into primary care and community-based platforms improves access to and continuity of care (P. Iqbal et al., 2022; Poudyal & Adhikari, 2022). Additionally, digital interventions expand service reach and enable culturally adapted language-sensitive support for displaced populations (Hynie et al., 2023).

Policy-level interventions emphasize national guidelines, inter-sectoral collaboration, resource allocation, and evaluation frameworks to ensure equity, quality, and adherence to PCC and human rights principles (Negash et al., 2025; Patel et al., 2018). Barriers to this include under-resourced systems, fragmented services, and inconsistent policy enforcement (Kirmayer & Jarvis, 2019). Facilitators include participatory service designs, provider training, and monitoring systems aligned with international guidelines.

## Discussion

While themes such as dignity, autonomy, and communication may appear in broader mental health literature, a PCC-specific perspective is necessary to systematically foreground these dimensions as core indicators of care quality rather than secondary outcomes (Rousseau, 2018). The PCC framework enables a normative, rights-based assessment of how mental health services are delivered and experienced, particularly within humanitarian settings where power asymmetries and structural constraints are pronounced.

The findings indicate that PCC-MH for migrants and refugees is not merely a set of interpersonal competencies, but a culturally negotiated process shaped by language, power, and normative assumptions embedded within humanitarian and host-country mental health systems. Cultural mismatch between providers’ biomedical frameworks and migrants’ explanatory models of distress emerged as a central mechanism undermining dignity, trust, and meaningful participation in care (Benjamen et al., 2021). Interventions such as culturally adapted cognitive behavioral therapy, value-based counseling, and culturally grounded assessment tools illustrate the ways in which aligning care with local meanings of distress can restore autonomy, reduce stigma, and enhance engagement (Grasser, 2022). Importantly, these cultural dynamics operate across multiple levels: at the micro level through communication and therapeutic relationships; at the meso level through service organization, interpreter availability, and cultural safety practices; and at the macro level through governance, funding priorities, and human-rights protections. These findings suggest that culture is not an adjunct to PCC-MH, but a foundational dimension through which person-centeredness is enacted, constrained, or enabled in humanitarian settings.

This review also confirms that refugees and asylum seekers, collectively categorized as displaced migrant populations, experience a substantially higher burden of mental disorders, including depression, anxiety, and PTSD, than host populations (Fazel et al., 2005; Priebe et al., 2016). This burden is particularly pronounced in humanitarian camps and low-resource settings, where material deprivation, insecurity, and protracted displacement compound pre- and peri-migration trauma (Desalegn, 2025). The absence of PCC approaches can restrict migrant voices and further hinder access to care throughout migration trajectories. Post-migration stressors such as legal precarity, discrimination, and social isolation further exacerbate mental health challenges (Meherali et al., 2024; Satinsky et al., 2019).

Evidence derived directly from humanitarian settings, particularly refugee camps and low-resource contexts, primarily demonstrates the magnitude of unmet mental health needs, the centrality of dignity, safety, and trust in care delivery, and the structural constraints affecting PCC-MH, including resource scarcity, legal precarity, and limited-service continuity. In contrast, much of the evidence informing specific PCC-MH implementation strategies, such as culturally adapted psychotherapies, interpreter-supported care, participatory service design, and provider training, is extrapolated from host-country and resettlement contexts (Tol et al., 2015). While these host-country studies provide useful and transferable principles, their applicability to humanitarian settings must be interpreted with caution, given differences in infrastructure, financing, and governance that may limit scalability and sustainability (Priebe et al., 2016).

Despite the high level of need, the utilization of mental health services remains consistently low, even in LMIC and humanitarian settings, due to structural barriers, stigma, and a lack of culturally competent care (Bischoff & Hudelson, 2010; Brandenberger et al., 2019). Vulnerable groups, including displaced migrant, women in the perinatal period, unaccompanied minors, and survivors of gender-based violence, face disproportionate risks and limited tailored interventions (Negash et al., 2025). Cultural and linguistic sensitivity is essential for effective person-centered care. Culturally adapted tools and interventions, such as the Afghan Symptom Checklist and value-based counseling, improve engagement, adherence, and outcomes, particularly in children and adolescents (Miller et al., 2006; Orang et al., 2023). Professional interpreters and provider training enhance communication and trust, while digital interventions offer opportunities to overcome access barriers if culturally adapted (Weissbecker et al., 2018).

Table 4 summarizes the key findings across the five PCC domains (dignity and supportive care, communication and autonomy, cultural and linguistic sensitivity, human-rights-based frameworks, and policy/system-level interventions), highlighting both the facilitators and barriers identified in the included studies. Findings represent synthesized patterns across included studies rather than results from individual studies.

**Table 4.** Synthesized Patterns of Person-Centered Mental Health Care (PCC-MH) Experiences Among Migrants and Refugees Across Key Domains.

PCC Dimension	Findings	Description
Dignity and supportive care	Positive experiences	Refugees and asylum seekers reported feeling respected, welcomed, and valued when providers demonstrated cultural empathy (Desalegn, 2025). Value-based counseling and culturally adapted cognitive behavioral therapy (CBT) enhanced trust and engagement (Hinton & Jalal, 2014; Orang et al., 2023).
	Negative experiences	Experiences of stigma, discrimination, neglect, and coercion were reported, particularly among women, unaccompanied minors, and survivors of gender-based violence (Brandenberger et al., 2019; Negash et al., 2025). Miscommunication and culturally insensitive care undermined trust and treatment adherence (Bischoff & Hudelson, 2010).
	Mixed experiences	Continuity of care and relational trust facilitated engagement; however, inconsistent provider cultural competence resulted in variable experiences of dignity and support (Canda, 2024; Lau & Rodgers, 2021).
Communication and autonomy	Positive experiences	Use of professional interpreters, co-designed interventions, and digital tools improved communication and patient participation (Hynie et al., 2023; Negash et al., 2025; Weissbecker et al., 2018). Refugees valued opportunities to express preferences and make informed decisions when culturally safe approaches were employed (Orang et al., 2023)
	Negative experiences	Language barriers, limited autonomy in decision-making, and lack of culturally adapted materials restricted meaningful participation (Bischoff & Hudelson, 2010; Kirmayer & Jarvis, 2019). Women and adolescents frequently reported restricted choice of services and limited empowerment (Negash et al., 2025).
	Mixed experiences	Engagement improved with culturally sensitive interventions; however, structural constraints such as understaffed facilities and legal restrictions continued to limit autonomy (Brandenberger et al., 2019; Priebe et al., 2016).
Cultural and linguistic sensitivity	Positive experiences	Culturally adapted assessment tools and therapies improved adherence, reduced drop-out, and enhanced clinical outcomes (Miller et al., 2006; Orang et al., 2023). Integration of local explanatory models and family systems supported child and adolescent mental health (Betancourt et al., 2013; Lustig et al., 2004).
	Negative experiences	Shortages of trained personnel, inconsistent institutional policies, and miscommunication hindered the delivery of culturally and linguistically sensitive care (Brandenberger et al., 2019; Weissbecker et al., 2018).
	Mixed experiences	Professional interpreters and peer-support mechanisms facilitated care, but implementation remained inconsistent across settings and contexts (Bischoff & Hudelson, 2010; Satinsky et al., 2019; Weissbecker et al., 2018).
Human rights and PCC frameworks	Positive experiences	Rights-based interventions enhanced dignity, autonomy, and equitable access to mental health services (Mezzina et al., 2018; Orang et al., 2023). Participatory and co-designed approaches strengthened therapeutic engagement and trust (Altahsh et al., 2025; WHO, 2023a)
	Negative experiences	Under-resourced health systems, discrimination, and inconsistent application of rights-based frameworks limited the effectiveness of PCC-MH (Brandenberger et al., 2019; WHO, 2024).
	Mixed experiences	When integrated with PCC, human-rights frameworks improved continuity of care; however, systemic and structural barriers persisted (Canda, 2024; Kronick et al., 2021; WHO, 2024)
Policy and system-level interventions	Positive experiences	Embedding PCC principles into policies, integrating mental health into primary care, and applying WHO frameworks improved service coordination and equity (Mezzina, 2025; WHO, 2018, 2024). Digitally enabled interventions expanded access when culturally adapted (Henley et al., 2011; Hynie et al., 2023).
	Negative experiences	Fragmented services, insufficient funding, and weak policy enforcement constrained PCC implementation (Brandenberger et al., 2019; Poudyal & Adhikari, 2022).
	Mixed experiences	Participatory service design and provider training supported PCC implementation, yet persistent system-level constraints limited sustainability and scale-up (Patel et al., 2018; Priebe et al., 2016).

Across PCC domains, this review demonstrates that migrants’ experiences of dignity, communication, autonomy, and cultural safety are shaped not only by interpersonal interactions with providers but fundamentally by broader structural and system-level determinants. Language barriers undermined autonomy by restricting informed decision-making and consent, while cultural mismatches reduced trust and acceptability of care. This demonstrates how micro-level experiences are mediated by meso-level service organizations and macro-level governance. Insecure legal status, limited entitlement to services, chronic underfunding, workforce shortages, and fragmented humanitarian and host-country systems further constrained the consistent implementation of rights-based PCC-MH; this resulted in a gap between normative frameworks and lived care experiences. These findings indicate that effective PCC-MH requires alignment between interpersonal practices and structural conditions, including legal protections, sustainable financing, and accountable governance mechanisms.

Human rights principles are integral to PCC, promoting dignity, autonomy, privacy, and equitable access (Mezzina et al., 2018; WHO, 2024). Rights-based frameworks and participatory services strengthen therapeutic engagement and continuity of care. However, persistent structural constraints, systemic discrimination, and under-resourced health systems continue to limit the full realization of PCC-MH for displaced populations (Brandenberger et al., 2019).

Policy and system-level interventions guided by the WHO frameworks (WHO, 2024) are critical for internationalizing PCC. Embedding PCC principles into organizational policies, integrating mental health into primary and community care, and implementing national guidelines enhance service coordination, equity, and sustainability. Key facilitators include provider training, participatory service design, and monitoring systems, whereas ongoing challenges include fragmented services, insufficient funding, and limited enforcement of policy guidelines (Patel et al., 2018; WHO, 2024).

## Implications and Recommendations

Theoretically, this review advances person-centred mental health care by reframing migrant mental health quality beyond service access toward lived experiences of dignity, autonomy, cultural safety, and human rights within structurally constrained humanitarian systems.

At the clinical level, the findings emphasize the need to strengthen the interpersonal and communicative dimensions of mental health care for migrants and refugees. Integrating professional interpreters and cultural mediators as core members of mental health teams is essential to ensure accurate communication, build trust, and reduce the risk of misdiagnosis (Fennig & Denov, 2021). Moreover, training providers in trauma-informed and culturally responsive person-centered care practices can enhance therapeutic relationships, support informed decision-making, and improve engagement and continuity of care in diverse humanitarian and host-country settings.

At the programmatic level, mental health services should be designed and implemented in partnership with refugee and migrant communities. Co-designing services with service users and community representatives can help ensure that interventions are culturally relevant, acceptable, and responsive to lived experiences. Embedding person-centered care indicators such as dignity, autonomy, and participation into routine program monitoring and evaluation frameworks can further support accountability, quality improvement, and the systematic assessment of service user experiences.

At the policy level, institutionalizing person-centered and human rights-based principles in national mental health policies is critical for sustainable implementation. By aligning humanitarian mental health programs with the WHO’s QualityRights implementation guidance, we can standardize rights-based practices across different settings, enhance governance and oversight, and ensure equitable access to mental health services for migrants and refugees. Such policy alignment can facilitate the transition from fragmented pilot initiatives to integrated, system-level approaches that uphold dignity, autonomy, and inclusion (WHO, 2019).

## Strengths and Limitations

This scoping review systematically synthesized evidence from diverse sources, applying a clear operational definition of PCC-MH care grounded in the WHO Quality Rights framework and adapted to migrant and refugee contexts. By including qualitative, quantitative, mixed-methods, and programmatic studies, it offers a comprehensive and rights-based overview of interventions and experiences while identifying critical evidence gaps in low-resource and humanitarian settings, particularly among vulnerable groups.

This scoping review has several limitations. First, inclusion was restricted to English-language publications, potentially excluding relevant evidence from non-English humanitarian contexts. Second, heterogeneity in study designs, populations, and outcome measures limited comparability and precluded formal quality appraisal. Third, evidence from low-resource humanitarian settings remains underrepresented, with many studies originating from high-income host countries. Finally, as a scoping review, the study maps existing evidence but does not assess intervention effectiveness or causality.

## Conclusion

This scoping review elucidates that migrants and refugees experience a disproportionately high burden of mental health challenges compounded by barriers such as language differences, cultural mismatches, stigma, and legal restrictions. Nonetheless, person-centered approaches that emphasize dignity, autonomy, cultural sensitivity, and human rights demonstrate a strong potential to enhance engagement, satisfaction, and mental health outcomes. Community-based and culturally adapted interventions, integration of interpreters and cultural mediators, trauma-informed care, and rights-based frameworks have all shown promise across various contexts. Nevertheless, implementation remains fragmented, with limited institutionalization and sustainability, particularly in low-resource humanitarian settings.

Embedding person-centered care principles into national health policies, integrating mental health into primary and community care systems, and promoting participatory service designs for migrants and refugees are essential steps toward equitable and dignified care. Future research should focus on developing longitudinal, context-specific models that bridge humanitarian and host-country systems, ensuring that mental health services for displaced populations are not only accessible but also uphold human rights and reflect the lived experiences of those they serve.

## Data Availability

The data that support the findings of this study are available from the corresponding author upon reasonable request.*
